# Whole-genome of Mexican-crAssphage isolated from the human gut microbiome

**DOI:** 10.1186/s13104-018-4010-5

**Published:** 2018-12-17

**Authors:** Melany Cervantes-Echeverría, Edgar Equihua-Medina, Fernanda Cornejo-Granados, Abigail Hernández-Reyna, Filiberto Sánchez, Blanca Estela López-Contreras, Samuel Canizales-Quinteros, Adrián Ochoa-Leyva

**Affiliations:** 1 Departamento de Microbiología Molecular, Instituto de Biotecnología, Universidad Nacional Autónoma de México, Avenida Universidad 2001, Colonia Chamilpa, 62210 Cuernavaca, Morelos Mexico; 2 Unidad de Genómica de Poblaciones Aplicada a la Salud, Facultad de Química, UNAM/Instituto Nacional de Medicina Genómica (INMEGEN), Ciudad de México, México

**Keywords:** crAssphage, Mexican-crAssphage, Human gut microbiome, Human phages, Host-microbiota

## Abstract

**Objectives:**

crAssphage is a newly found phage described as the most abundant virus in the human gut microbiome. The majority of the crAssphage proteins are unknown in sequences databases, and its pathogenicity and epidemiology in humans are yet unclear. Hence, being one of the most abundant phages in the human gut microbiome more investigation at the genomic level is necessary to improve our understanding, especially in the Latin American population.

**Data description:**

In this article, we provide the whole genome of a crAssphage isolated from the human gut microbiome of the Mexican population, which was named Mexican-crAssphage. The genome consists of 96,283 bp, G+C content of 29.24% and 87 coding sequences. Notably, we did not find any transfer RNA genes in the genome sequence. We also sequenced viral-like enriched particles from 28 fecal samples, and we detected the presence of the Mexican-crAssphage genome in 8 samples (28.5%). To our knowledge, our data is the first whole genome report of the crAssphage isolated from the Latin American Population and provides valuable information for the experimental characterization of the most abundant human gut bacteriophage. The whole genome shotgun project of the Mexican-crAssphage is available at DDBJ/ENA/GenBank under the GenBank MK069403.

## Objective

The human body is inhabited by a high diversity of bacteria, archaea, fungi, protozoa, and viruses. These microbes are collectively known as the human microbiota, whereas their collective genomes form the human microbiome [1]. The human gut virome is dominated by bacteriophages [2], infecting their bacterial hosts and they also impact the microbiome composition [1]. Interestingly, it has been proposed that bacteriophages may have a role in shaping the diversity and composition of the microbiota [1] and also play a role in some diseases such as bowel disease [3] and type 1 and 2 diabetes [4, 5].

A novel bacteriophage, named crAssphage, was recently discovered as the most abundant virus in the human gut microbiome [6]. After that, a crAss-like family was discovered and appears to be abundant and widespread in diverse habitats, both animal and environmental associated [7]. Various bacteria of the phylum Bacteroidetes appear to be the primary hosts of crAss-like phages [6, 7]. For example, ΦCrAss001, isolated from the human feces, was the first member of the extensive crAssphage family to be grown in pure culture and this phage infects the human gut symbiont *Bacteroides intestinalis* [8]. Recently, 98 complete circular genomes of crAss-like phages were reported and helped to establish the classification of this phage family into four candidate subfamilies composed of 10 candidate genera [9]. Furthermore, crAssphage was not associated with diarrhea in Chinese patients [10]. The crAssphage genomes have been isolated from the human gut of several geographical origins (Data file 1 in Table 1). However, a genome sequence from this phage family has not been reported to date in a Latin American population. Hence, being one of the most abundant phages in the human gut microbiome more investigation at the genomic level is necessary to improve our understanding about their function, especially in the Latin American population.

**Table 1:** Overview of data files/data sets

Label	Name of data file/data set	File types (file extension)	Data repository and identifier (DOI or accession number)
Data set 1	Whole genome	FASTA file	GenBank Accesion number: MK069403 (https://www.ncbi.nlm.nih.gov/nuccore/MK069403)
Data file 1	Geographical origin of the crAssphage genomes previously reported	MS Excel file (.xslx)	Figshare (10.6084/m9.figshare.7379600)
Data file 2	Read depth and proteins along the Mexican-crAssphage genome.	Adobe Portable Document Format (.pdf)	Figshare (10.6084/m9.figshare.7379603)

## Data description

Phage-enriched filtrates of fecal samples from 28 Mexican children were isolated using a modified protocol [11]. In brief, 250 mg of feces were homogenized in SM Buffer for each sample and centrifuged 30 min at 4700×*g*. The supernatant was filtered through a 0.22 μm PES filter (720–1320, Nalgene, USA) and concentrated in Amicon Ultra 15, 100KDa (UFC910096, Millipore, USA). Then, Amicon was washed using one volume of SM Buffer, and the viral particles were concentrated in 200 µl of SM buffer. We extracted the DNA using the QIAamp MinElute Virus Spin kit (57704, QIAGEN, Hilden, Germany). The DNA quality and quantity were measured using agarose gel electrophoresis and Qubit High-sensitivity fluorometric assay (Cat. Q32851, Life Technologies, Carlsbad, CA, USA), respectively. The DNA was used to construct the pair-end libraries using the Nextera XT DNA Library Preparation kit (Cat. FC-131-1024, Illumina, CA, USA) selecting an insert size of 400–600 bp with the Ampure XP beads (Cat. A63882, Beckman Coulter, CA, USA). The libraries were analyzed with the 2100 Bioanalyzer instrument (Cat. 5067-1504, Agilent Technologies, CA, USA), and sequencing was performed using the Illumina NextSeq500 with a 300 cycle paired-end format (FC-404-2003; Illumina, CA, USA) at the National Institute of Genomic Medicine in Mexico City. The reads were analyzed using FastQC version 0.11.5, and only the reads with a quality > Q20 were used for further analysis. The resulting reads from each sample were mapped against the crAssphage reference genome (GenBank ID: JQ995537) using SMALT version 0.7.6. After that, we selected the sample with the highest number of reads mapped to crAssphage genome to conduct a denovo genome assembly using Spades version 3.8.1. The resulting contigs were ordered using MeDuSa [12] setting the default parameters.

The total size of the assembled Mexican-crAssphage genome was 96,283 bp and G+C content of 29.24% (Data set 1 in Table 1). The reads coverage of our Mexican-crAssphage genome was 188X. To visualize the read depth and codified proteins along the Mexican-crAssphage genome we used DNAPlotter (Data file 2 in Table 1). A total of 87 coding sequences (CDS) were predicted using RAST [13]. They were largely co-oriented, organized in two blocks of CDS alongside the genome. These sequences were BLASTed against the non-redundant (NR) proteins database using Blast2GO [14]. After that, 12 proteins (13.8%) were unknown, and 60 proteins (68.9%) were defined as hypothetical protein. The genome showed to encode phage proteins, including proteins involved in nucleic acid manipulation (helicase, ligase, primase, and polymerase), and phage structural proteins. Notably, we did not find any transfer RNA genes in the genome sequence.

Finally, the viral reads of the 28 samples were mapped against the Mexican-crAssphage genome using SMALT version 0.7.6. We detected the presence of this phage’s genome in eight samples, meaning that the Mexican-crAssphage was present in 28.5% of the analyzed samples. This is the first CrAssphage genome isolated from a Latin-American population, and it can be used in different applications of human viral metagenomics to understand the impact that host-genetics have in modulating the evolution of crAssphage across the world.

## Limitations

A more significant deep sequencing of viral particles should be used in the future to improve a region of 100 uncalled bases (N’s) reported in this Mexican-crAssphage genome. This region is in positions 40,116–40,215 of the reported genome. It is important to note that these are the only missing bases from all the genome.
